# Seroprevalence and risk factors for brucellosis amongst livestock and humans in a multi-herd ranch system in Kagera, Tanzania

**DOI:** 10.3389/fpubh.2024.1478494

**Published:** 2024-11-01

**Authors:** Beatus Lyimo, Ephrasia Hugho, Coletha Mathew, Charles Mayenga, Abdul Hamid Lukambagire, Samson Lyimo, Lidia Munuo, Maurice Byukusenge, Jodie Withall, Roland T. Ashford, Blandina T. Mmbaga, Zachariah Makondo, John McGiven, Jessica Radzio-Basu, Erika Ganda, Earl A. Middlebrook, Andrew W. Bartlow, Jeanne M. Fair, Gabriel Shirima, Nammalwar Sriranganathan, Rudovick R. Kazwala, Peter J. Hudson, Isabella M. Cattadori, Vivek Kapur, Joram J. Buza, Robab Katani

**Affiliations:** ^1^Nelson Mandela African Institution of Science and Technology, Arusha, Tanzania; ^2^Kilimanjaro Clinical Research Institute, Moshi, Tanzania; ^3^Kilimanjaro Christian Medical University College, Moshi, Tanzania; ^4^Sokoine University of Agriculture, Morogoro, Tanzania; ^5^Tanzania Veterinary Laboratory Agency, Dar es Salaam, Tanzania; ^6^Department of Veterinary and Biomedical Sciences, Pennsylvania State University, University Park, PA, United States; ^7^Department of Bacteriology, Animal and Plant Health Agency, Weybridge, United Kingdom; ^8^Department of International Development, Innovation and Business, Animal and Plant Health Agency, Weybridge, United Kingdom; ^9^Huck Institutes of the Life Sciences, Pennsylvania State University, University Park, PA, United States; ^10^Department of Animal Science, Pennsylvania State University, University Park, PA, United States; ^11^Los Alamos National Laboratory, Los Alamos, NM, United States; ^12^Virginia-Maryland Regional College of Veterinary Medicine, Virginia Tech, Blacksburg, VA, United States; ^13^Department of Biology, Pennsylvania State University, University Park, PA, United States

**Keywords:** brucellosis, Tanzania, zoonotic, livestock, public health, low-middle-income countries

## Abstract

**Background:**

Brucellosis remains a significant health and economic challenge for livestock and humans globally. Despite its public health implications, the factors driving the endemic persistence of *Brucella* at the human-livestock interface in Tanzania remain poorly elucidated. This study aimed to identify the seroprevalence of *Brucella* infection in livestock and humans within a ranching system and determine associated risk factors for disease endemicity.

**Methods:**

A cross-sectional sero-epidemiological study was conducted in 2023 in Tanzania’s Karagwe District, involving 725 livestock (cattle, goats, sheep) from 10 herds and 112 humans from associated camps. Seroprevalence was assessed using competitive ELISA while epidemiological data were collected via questionnaires. Generalized Linear Models and Contrast Analysis were used to identify risk factors for infection.

**Results:**

Overall seroprevalence was 34% in livestock and 41% in humans. Goats exhibited the highest prevalence (69.2%), while cattle had the lowest (22.6%). Mixed-species herds (Odds Ratio, OR = 2.96, CI [1.90–4.60]) and small ruminants-only herds (OR = 6.54, CI [3.65–11.72]) showed a significantly higher risk of seropositivity compared to cattle-only herds. Older cattle (OR = 5.23, CI [2.70–10.10]) and lactating females (OR = 2.87, CI [1.78–4.63]) represented significant risks for brucellosis in livestock. In humans, close contact with animals (OR = 7.20, CI [1.97–36.31]) and handling animals during parturition or aborted fetuses (OR = 2.37, CI [1.01–5.58]) were significant risk factors. Notably, no spatial association was found in seroprevalence between herds and nearby human communities.

**Conclusion:**

The lack of spatial correlation between livestock and human seroprevalence suggests complex transmission dynamics, potentially involving endemic circulation in livestock and human infections from multiple sources of exposure to livestock. This study highlights the need for comprehensive zoonotic risk education and targeted intervention strategies. Further research is crucial to elucidate transmission pathways and improve *Brucella* infection control. This includes developing robust methods for identifying infective species and implementing effective strategies to mitigate *Brucella* infection in endemic regions.

## Introduction

Zoonotic infections, particularly those endemic in livestock and domestic reservoir animals, pose significant global public health challenges. Brucellosis, caused by bacteria of the genus *Brucella*, exemplifies these challenges, especially at the human-animal interface in low- and middle-income countries (LMICs). According to the World Health Organization (WHO), brucellosis is one of the most widespread zoonoses with serious public health consequences in endemic areas (1). The global human incidence is estimated at 1.6–2.1 million new cases annually, with a disproportionately high prevalence in LMICs (2). Furthermore, a 2011 World Bank report identified brucellosis as one of the top five causes of infectious disease losses in cattle, buffalo, sheep, goats, and camels, highlighting its significant economic impact on animal agriculture (3). The control and prevention of brucellosis, like many zoonoses, are hindered by inadequate surveillance, limited access to reliable diagnostics, and insufficient understanding of risk factors (4, 5). These challenges underscore the need for a One Health approach in addressing brucellosis and similar zoonotic diseases.

Despite the public health importance and the substantial economic burden on farmers, brucellosis is underreported due to the non-pathognomonic nature of its symptoms, which may be similar to malaria in humans (6). The disease is now reported as endemic in many livestock populations from LMICs (7, 8). In rural settings, where dependence on agriculture and livestock is frequently the sole source of livelihood (9, 10), infections can spread rapidly in animals and people due to limited surveillance and limited understanding of the risk factors for infection (11).

In livestock, brucellosis is primarily contracted via exposure to infected animals’ bodily fluids, particularly those associated with parturition or abortion, ingestion of contaminated food and water, or vertical transmission from mother to offspring during pregnancy or parturition. The infection causes abortion, stillbirth, and reduced productivity in animals (12). Human infection occurs through the consumption of unpasteurized dairy products or direct contact with infectious animal fluids and tissues, often as occupational exposure in professions such as veterinarians, livestock handlers, or abattoir workers (13).

Tanzania holds the third largest livestock population in Africa (14), and faces significant challenges with endemic brucellosis (14). Outbreaks have been documented since 1927 (15), although prevalence data are scattered and show considerable variation among regions and over time (14, 16, 17). For example, seroprevalence in livestock can vary from 1 to 46% (8, 18–22), while in human populations it has been reported from 0.6 to 58.4% (12, 17, 23–25). This variation may be due to ecological differences in the host-pathogen interaction and disease transmission, variation in livestock management, but could also be the result of inadequate surveillance and control interventions, or a combination of all these factors.

As reported by the Tanzania Livestock Modernization Initiative, the majority of livestock management in Tanzania falls under the traditional agro-pastoral (80% of the livestock population) and pastoral (14%) systems (26). The remaining 6% is attributed to non-traditional, commercial ranching, and dairy sectors. While research on brucellosis in this country has predominantly concentrated on the pastoral and agro-pastoral systems, there remains a significant gap in knowledge regarding the incidence and impact within the commercial ranching sector.

To address this gap, our study aimed to quantify the seroprevalence of brucellosis in 10 livestock herds, comprising a population of 7,000 livestock, and associated human camps from a 12,000-acre commercial ranch in Tanzania’s Kagera region. Specifically, we sought to: (1) Identify the pathogen seroprevalence in herds of different structure and composition (e.g., animal species, age and size); (2) Determine how seroprevalence from individual herds is associated with the prevalence of infection in the local human community. We then used this information and questionnaires to these human communities to: (3) Identify the risk factors of brucellosis in both humans and livestock.

By elucidating these factors within a ranch system, our study provides insights that can help to improve livestock management and productivity in similar settings, ultimately contributing to better control of this important zoonotic disease.

## Materials and methods

### Ethics approval and consent to participate

The study protocol was approved by Pennsylvania State University, IACUC-PSU (PROTO202101993), and IRB-PSU (STUDY00018709). The study was also approved by the National Institute for Medical Research (NIMR/HQ/R.8c/Vol. IX/3636) and the Institutional Ethics Approval for Kilimanjaro Christian Medical University College Research Ethics and Review Committee (CRERC No: 2494) in Tanzania. The research permission was also received from the President’s Office, Regional Administrative and Local Government (PO-RALG Ref. AB 307/223/01) of Tanzania and the Regional administrative secretary of the participating regions in Tanzania. The research was performed in accordance with the relevant guidelines and regulations prescribed by the above research regulatory committees. For human subjects, written informed consent was sought and obtained from all the adult participants (18 years and above).

### Study site and period

**Figure 1:**
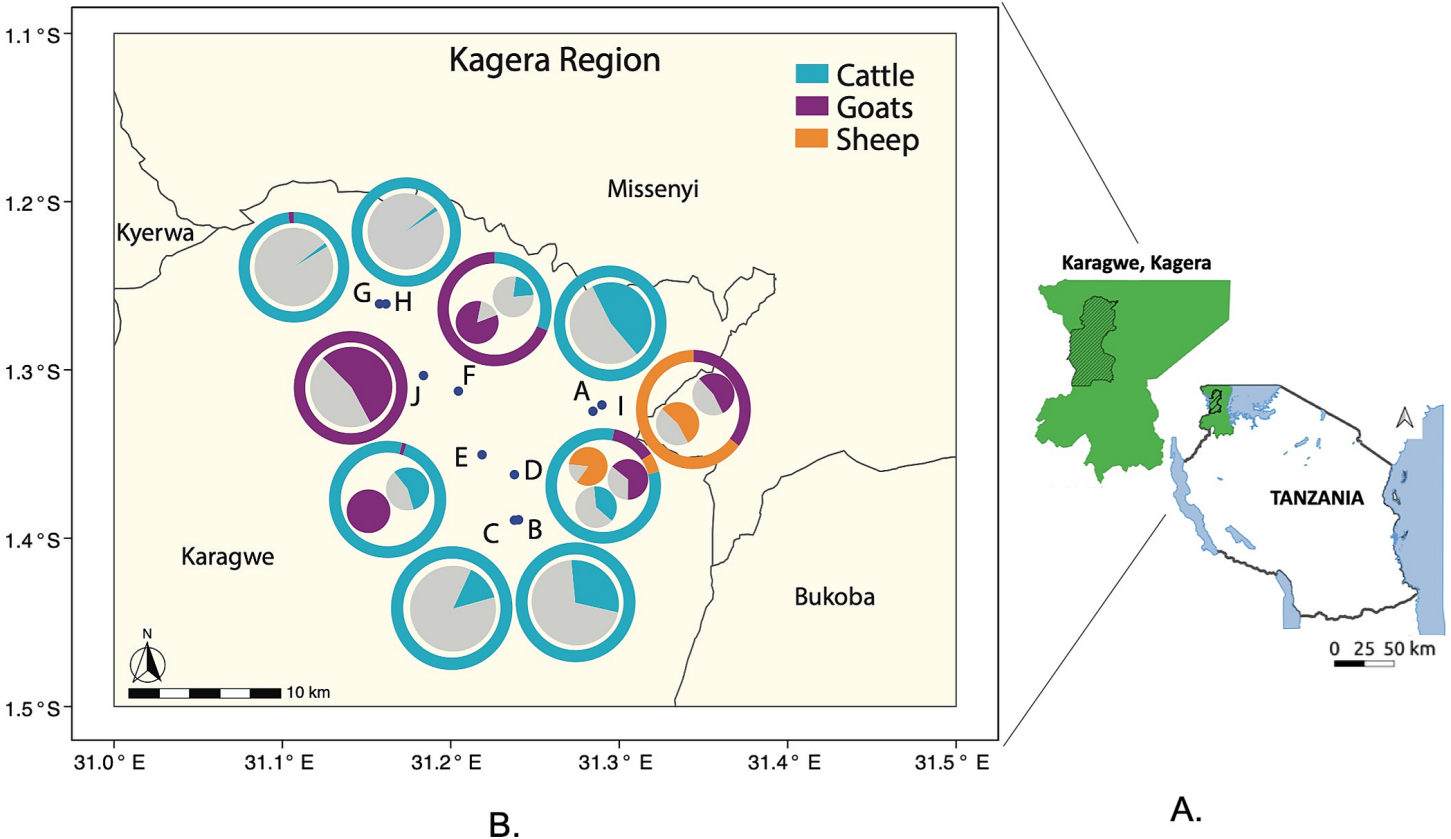
Location and composition of the 10 sampled herds within the Kagera region ranch. **(A)** Map of Tanzania and the sampling region (Karagwe District of Kagera region in green slash area, Kagera region in green shade). **(B)** Each herd (A-J) is represented by a point on the map. The herds are labeled A through J, for a total of 10 herds. The outer donut graph: Around each point (herd location) is a donut-shaped graph. These graphs show the species composition of each herd as percentages, and the species are represented by different colors. The inner pie graphs: Inside each donut graph is a smaller pie graph. These pie graphs show the percentage of animals that tested seropositive for brucellosis within each species in the herd. The colors represent different species: Blue represents cattle, Purple represents goats, and Orange represents sheep.

Field sampling was undertaken in January 2023 in a commercial ranch in the Lake zone of the Kagera region of Tanzania (Figure 1). Kagera is situated in the northwestern part of the country and shares borders with Uganda, Rwanda, and Burundi, and experiences a tropical climate characterized by two rainy seasons and an annual average temperature of 21°C (27). These climatic conditions facilitate the development of agricultural activities, such as livestock farming and crop cultivation.

### Livestock and human populations

The ranch system surveyed for this study manages about 7,000 livestock making it a pivotal regional livestock enterprise. Livestock are organized into a total of 13 herds of single- or mixed-species indigenous cattle, sheep, and goats (Supplementary Table S1). Herds can be considered independent units with their own livestock management, where mixing between neighboring herds is uncommon, except on special occasions or events such as droughts when animals are forced to congregate at watering points. The human community working and living on the ranch are organized in camps, and individuals move freely between camps while working on different herds, thus the spatial organization of the human community is partially independent of the herd system.

### Epidemiological survey

To assess brucellosis seroprevalence, we conducted a cross-sectional study involving both livestock and humans associated with a ranch in Tanzania. We randomly selected herds and the human community from camps these herds. For every herd, a minimum of 10% of the total animals was opportunistically selected for sampling.

### Sample size calculation

To determine the appropriate sample size, we employed, Cochran’s Sample Size approach (28) to provide an *a priori* estimate of the required number, adjusted for smaller populations. The formula used was: n_ajd_ = (Nn_o_)/(N+ n_o_), where n_o_ = (z^2^p (1 – p))/d^2^. For our calculations, we assumed a total ranch population (N) of 7,000 animals, an expected prevalence (*p*) of 8.2% based on pooled prevalence data from previous studies in the area (14, 29–33), a precision level (d) of 0.02, and a confidence level of 95% (Z = 1.96). The required sample size was estimated to be 721 total animals of 6 months or older. For the human population, we targeted individuals from camps associated with the sampled herds. Using the same approach as for livestock, we calculated a required sample size of 108 adults. This calculation was based on an estimated adult population of 250 and an expected prevalence of 2.1%, derived from pooled prevalence data in the area (14, 17, 34).

### Data collection methods

Data collection was facilitated through the use of EpiCollect (35), a digital data-gathering platform. For animals, we recorded species, age, sex, identification number, GPS coordinates, and lactation status. Human participants provided information on age (≥18 years), gender, occupation, raw meat/milk consumption, and engagement in high-risk activities associated with brucellosis. To ensure anonymity, each human participant was assigned a unique, de-identified code.

### Questionnaire administration

To maximize comprehension and accuracy of responses, questionnaires were provided to human participants in both English and Swahili. Additionally, local research assistants fluent in both languages were available to assist participants and address any questions or concerns that arose during the data collection process. This comprehensive sampling and data collection approach allowed us to obtain a representative sample of both livestock and humans within the ranch system, providing a holistic view of brucellosis seroprevalence in this setting.

### Blood sample collection

Blood specimens were collected from livestock and humans who agreed to participate in the study. For livestock, following skin disinfection with 70% alcohol, blood was drawn aseptically by the veterinarian from the jugular vein into empty 5 mL Vacutainer tubes (BD Vacutainer, UK). Samples were allowed to coagulate at ambient temperature for about 6 h, and serum was then separated from clotted blood and transferred to 1.8 mL screw cryovial tubes (Thomas Scientific, USA). For human subjects, following skin disinfection with isopropyl alcohol and povidone-iodine, 5 mL of blood was aseptically taken by registered nurses from the brachial vein using the same type of tubes and subsequent serum isolation protocol. Both livestock and human sera were transported to the final testing laboratories in vehicle freezers and stored at −20°C before processing.

### Laboratory procedures

#### Enzyme-linked immunosorbent assay (cELISA)

The preliminary identification of serum immunoglobulin against *Brucella* was based on the Rose Bengal Plate Test (36). The Rose Bengal Test (RBT) was initially employed to identify this ranch as a potential area of interest due to its suitability for rapid, field-based screening. However, for laboratory testing, the competitive Enzyme-Linked Immunosorbent Assay (cELISA) was subsequently chosen for its higher specificity and efficiency in confirming *Brucella* seroprevalence. Therefore, the presented results focus exclusively on the cELISA findings for evaluating risk factors and the statistical analyses.

The commercially available competitive enzyme-linked immunosorbent assay (c-ELISA) Svanovir *Brucella*-Ab c-ELISA (37) was used to detect the presence of specific antibodies to *Brucella,* according to the manufacturer’s instructions. Briefly, 5 μL of diluted serum samples and kit-provided ready-to-use controls were added to the microtiter plate wells containing the antigen. The assays were then incubated at 25°C for 30 min, after which the first wash was performed. Later, conjugate with an enzyme was added and incubated for 30 min at 37°C. All wells were washed to remove excess conjugate, followed by a new incubation for 30 min at 37°C with the enzyme substrate. Finally, the reaction was interrupted by adding 100 μL of the stopping solution. Samples were quantified using optical density (OD) measured at 450 nm in a microplate photometer. Specifically, we followed the kit’s protocol and calculated the percent inhibition (PI) values for controls and samples as: PI = 100-(OD_sample or control_/OD_Conjugate Control_) × 100. To ensure validity, the values of the controls must fall within the following limits: OD Conjugate Control (Cc) 0.75–2.0; PI Positive control: 80–100; PI Weak Positive control: 30–70; PI Negative control <30. If all control values were within acceptable ranges, the status of a test sample was determined as follows: If the PI status was <30%, the sample was considered Negative, and if the PI was ≥30%, the sample was considered Positive.

#### Malaria testing

Malaria testing was performed on all human sera to rule out potential confounding effects caused by this infection, as malaria symptoms can mimic those of brucellosis in endemic areas. Whole blood samples were tested with the SD Bioline Malaria Antigen test (BioLine™ Malaria Ag P.f/Pan test, Standard Diagnostics Ref 05FK60, Inc.; Suwon City, Republic of Korea) for the detection of histidine-rich protein 2 antigens of *P. falciparum* and *Plasmodium* lactate dehydrogenase (pLDH) specific to plasmodium, according to the manufacturer’s instructions. Briefly, 5 μL of whole blood was dispensed into the round specimen well, and four drops of assay diluent (∼30 μL) into the assay well; results were interpreted in 15–30 min.

### Statistical analysis

To examine whether the number of ELISA seropositive cases in livestock was significantly different by herd and animal characteristics, binomial logistic regressions were performed on cross-sectional data using Generalized Linear Model (GLM with family = binomial) in the statistical software R (v. 4.1.2) (38).

The analysis followed three main steps. First, the original animal data were split into two sets: 85% were randomly allocated to the training set and the remaining 15% to the testing set. Second, the training set was used to fit the model of interest, and the goodness of the fit was estimated according to the Akaike Information Criterion (AIC) (39). Specifically, the training set was used to investigate if the probability of animal seropositive cases (1 = positive, 0 = negative), as a response variable, was affected by specific herds (1–10) or their size, livestock species (cattle, goats, and sheep) or animal characteristics (age, sex, and lactation status), as independent variables. This was performed using univariate GLMs, i.e., one independent variable at a time with variables included as categorical except herd size; the estimated AIC provided the goodness of each model fit. In the third step, the testing set was used to examine the performance of models selected at step two by estimating their ability to provide similar predictions using a different dataset (40). A model that well explains the association between a predictor variable and an outcome variable, using the training data, and that does equally well when applied to a different data set is evidence of a strong predictor and thus is an important biological association. A probability threshold of *p* = 0.5 was chosen, where *p* > 0.5 indicated a positive result (1), and *p* ≤ 0.5 indicated a negative result (0). The general predictive ability of the model examined on the testing data set was visually investigated by plotting the ROC curve (Receiver Operating Characteristics) using the package *ROCR* in R (41). This curve describes the relationship between the true positive rate and the false positive rate at various threshold, p, settings. The area under the ROC curve (AUC) was calculated where an AUC close to 1 indicates strong model performance, meaning a greater ability of the model (selected using the training set) to correctly predict positive cases as 1 and negative cases as 0 from the testing dataset. Pairwise *post-hoc* Contrast analysis [R package *emmeans*, (42)] was carried out for the categorical variables with more than two factors, based on the estimated marginal means adjusted for the other factors in the model. Finally, to facilitate the interpretation of the results, the estimated coefficients from GLMs and Contrast analyses were converted to Odd Ratios (or adjusted Odd Ratios) (OR) by exponentiating the values of these coefficients and presenting the results as ORs throughout our work.

This same general approach was also applied to examine human ELISA data. As independent variables, we considered general demographic information (i.e., age, gender, education), and human activities or behaviors associated with livestock and where exposure to animal and/or animal products are considered at risk for *Brucella*. For livestock activities we considered two variables: ‘Occupation,’ which included herding, milking, feeding, providing animal health care (i.e., veterinarians), and ‘Handling,’ namely helping animal parturition, handling aborted fetuses or slaughtering. For behavior we used the variable ‘Habit’ that comprised the consumption of raw meat and milk; all these variables were included as categorical.

## Results

### Livestock serology

Overall, we collected 725 blood samples from livestock within the ten herds (Figure 1). Most of the samples were from cattle (74.5%), followed by goats (21.9%) and sheep (3.6%) (Table 1). The largest number of samples were collected from Herd G (16.9%) and the fewest from Herd C (3.7%). The majority of the collected samples were obtained from females, comprising 88.4% of the total sample population. Specifically, Herds A, G, H, and J were exclusively represented by female animals. It is noteworthy that the sampling process was conducted randomly, yet it resulted in a predominance of female samples. Most of the animals sampled were 2 years and older (87.3%), while the remaining 12.7% were 7 months to 2 years old. Among the females tested, 40.9% were lactating, specifically 53.8% cattle and 46.2% goats, while none of the sampled sheep were lactating. The comprehensive livestock demography is detailed in Table 1.

**Table 1:** Summary of seroprevalence data based on serum ELISA analysis from livestock.

Livestock risk factors	Cattle		Goats		Sheep	
	N (Total, positive)	Seroprevalence 95% [CI]	N (Total, positive)	Seroprevalence 95% [CI]	N (Total, positive)	Seroprevalence 95% [CI]
A. Herd ID (% Herd-level seroprevalence)						
Herd A (46.3%)	(41, 19)	46.3 [31.08–61.61]	0	–	0	–
Herd B (29.6%)	(27, 8)	29.6 [12.41–46.85]	0	–	0	–
Herd C (13.8%)	(29, 4)	13.8 [1.24–26.34]	0	–	0	–
Herd D (43.8%)	(92, 35)	38.0 [28.12–47.96]	(14, 9)	64.3 [39.19–89.39]	(6, 5)	83.3 [53.51–113.15]
Herd E (56.6%)	(82, 46)	56.1 [45.36–66.84]	(1, 1)	100.0	0	–
Herd F (64.4%)	(28, 6)	21.4 [6.23–36.63]	(62, 52)	83.9 [74.72 – 93.03]	0	–
Herd G (1.7%)	(121, 2)	1.6 [0.62–3.92]	(2, 0)	0	0	–
Herd H (1.7%)	(120, 2)	1.7 [0.62–3.96]	0	–	0	–
Herd I (54.8%)	0	–	(11, 6)	54.5 [25.12–83.97]	(20, 11)	55.0 [33.2–76.8]
Herd J (60.9%)	0	–	(69, 42)	60.9 [49.35–72.39]	0	–
B. Age						
7M-2 YRS	(39, 8)	20.5 [7.84–33.19]	(48, 33)	68.8 [55.64–81.86]	(5, 1)	20 [−15.06–55.06]
>2 YRS	(501, 114)	22.8 [19.08–2 6.43]	(111, 77)	69.4 [60.79–77.94]	(21, 15)	71.4 [52.11–90.75]
C. Sex						
Female	(489, 112)	22.9 [19.18–26.63]	(141, 100)	70.9 [63.43–78.42]	(11, 6)	54.5 [25.12–83.97]
Male	(51, 4)	7.8 [0.46–15.22]	(18, 10)	55.6 [32.6–78.51]	(15, 10)	66.7 [42.81–90.52]
D. Lactation status						
Yes	(141, 54)	38.3 [30.27–46.32]	(121, 90)	74.4 [66.6–82.16]	0	0
No	(348, 58)	16.7 [12.75–20.58]	(20, 10)	50 [28.09–71.91]	(11, 6)	54.5 [25.12–83.97]
Total	(540, 122)	22.6 [19.07–26.12]	(159, 110)	69.2 [62.01–76.36]	(26, 16)	61.54 [42.84–80.24]

Overall, the Enzyme-Linked Immunosorbent Assay (ELISA) seropositivity from the tested livestock was 34.2%. If we consider the herds, the highest seropositivity was recorded in Herd F (64.4%), a mixed herd composed mostly of goats (68.9%) and cattle (31.1%), while the lowest value was found in the cattle-only herds, namely Herds G and H (both at ~1.7%) (Table 1). Among species, goats exhibited the highest seroprevalence (69.2%, CI [62.01–76.36%]), followed by sheep (61.54%, [42.84% – 80.24]) and cattle (22.6%, CI [19.07–26.12%]) (Table 1). Logistic regressions were conducted to identify whether seropositivity was associated with herd ID, animal species or herd composition or size, independently for each of these variables (Table 2). ELISA seropositivity varied considerably among herds. The adjusted odds ratios of Herds C (OR = 0.24 CI [0.07–0.82]), G (OR = 0.02 CI [0.005–0.10]) and H (OR = 0.02 CI [0.005–0.11]) were significantly lower than 1, when compared to the reference Herd A, indicating a relatively lower risk of detecting a seropositive animal in these herds (Table 2A). The remaining herds showed both positive and negative adjusted ORs although not statistically significant when compared to the reference Herd A. The ability of this model, estimated on the training dataset, to provide similar predictions using the tested dataset showed both strong predicted accuracy (0.79) and performance (AUC = 0.87). This supports the important role of herds in explaining the variability of livestock seroprevalence observed. The selection of Herd A as the reference entity in the model was arbitrary and, in our case, simply based on the alphabetical order of the herds. Importantly, changing the reference herd does not change the general conclusion of our results, other than rescaling the ORs to a different herd. Given the relatively high seropositivity of Herd A (46.3%), it is not surprising that half the herds had an adjusted OR of less than one – three of which were significantly less than 1. Similarly, the adjusted OR of four herds were larger than one, confirming their higher, albeit not significant, seroprevalence when compared to Herd A (Tables 1, 2). To obtain a pairwise comparison between herds, a *post-hoc* Contrast analysis was carried out. Results substantiated the large variation in seroprevalence between herds, specifically, Herds C, G and H were significantly different when compared to the other herds, while the remaining herds were more heterogeneous in the number of significant pairwise comparisons (Supplementary Table S2).

**Table 2:** Logistic regression between serum ELISA (positive–negative) and potential risk factors for livestock.

Risk factors	OR	95% CI	*p*-value
A. Herds (Herd A Reference)			
Intercept	0.85	0.45–1.62	0.62
Herd B	0.63	0.21–1.84	3.95E-01
**Herd C**	**0.24**	**0.07–0.82**	**2.40E-02**
Herd D	0.95	0.44–2.03	8.87E-01
Herd E	1.47	0.66–3.26	3.43E-01
Herd F	2.03	0.92–4.48	8.01E-02
**Herd G**	**0.02**	**0.00–0.10**	**1.20E-06**
**Herd H**	**0.02**	**0.01–0.11**	**2.44E-06**
Herd I	1.39	0.50–3.90	5.31E-01
Herd J	1.67	0.73–3.83	2.29E-01
AIC = 602.89; Accuracy = 0.79; Performance (AUC) = 0.87			
B. Species (Cattle Reference)			
**Intercept**	**0.30**	**0.25–0.378**	**<2E-16**
**Sheep**	**6.57**	**2.59–16.70**	**7.56E-05**
**Goats**	**6.80**	**4.46–10.39**	**<2E-16**
AIC = 702.34; Accuracy = 0.79; Performance (AUC) = 0.76			
C. Herd composition (Cattle only Reference)			
**Intercept**	**0.21**	**0.14–0.30**	**9.78E-16**
**Cattle + small ruminants**	**2.96**	**1.90–4.60**	**1.55E-06**
**Small ruminants only**	**6.54**	**3.65–11.72**	**2.88E-10**
AIC = 750.80; Accuracy = 0.72, Performance (AUC) = 0.75			
D. Age (7 m-2 yrs. vs. >2 yrs)			
**Intercept**	**0.08**	**0.05–0.15**	**2.41E-15**
**Cattle**	**5.23**	**2.70–10.10**	**8.80E-07**
AIC = 478.02; Accuracy = 0.82; Performance (AUC) = 0.65			
E. Animal lactation (Yes vs. No)			
**Intercept**	**0.20**	**0.14–0.27**	**<2E-16**
**Cattle**	**2.87**	**1.78–4.63**	**1.49E-05**
AIC = 432.11; Accuracy = 0.74; Performance (AUC) = 0.66			
**Goats**	**3.65**	**1.16–11.50**	**2.67E-02**
AIC = 140.40; Accuracy = 0.69; Performance (AUC) = 0.42			

The Adjusted Odds Ratios (OR), Confidence Intervals (CI), Statistical Significance (*p* < 0.05), Goodness of model fit (AIC), from the model fitted to the training dataset, and Model Accuracy and performance [i.e., Area Under the Curve (AUC)] from the tested dataset, are reported. The intercept represents the reference entity. The significant attributes are represented in Bold.

**Figure 2:**
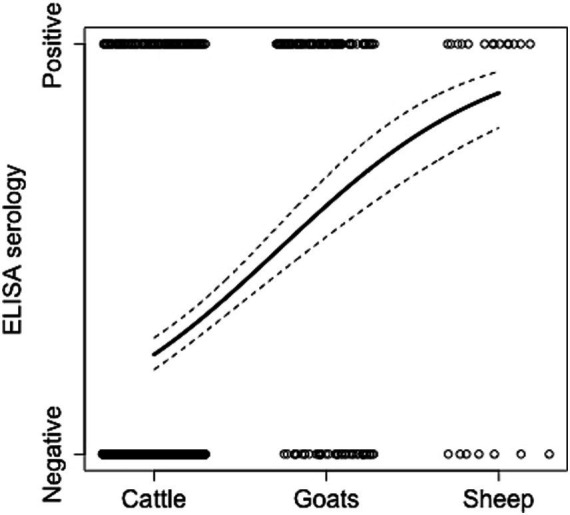
Relationship between ELISA cases and livestock species. The animal status (positive or negative to *Brucella*) from the collected samples (empty circles), the estimated model prediction (thick line), and related lower and upper 95% Wald confidence intervals (dashed lines) from the logistic regression using the trained data set are reported (full results of this analysis are in Table 2B). Circles are jittered to facilitate the visualization of every animal sampled in every herd.

We then examined if variation in ELISA seropositivity was related to livestock species. Goats exhibited the highest significant odds ratio (OR = 6.80, CI [4.46–10.39]) followed by sheep (OR = 6.57, CI [2.59–16.70]), when compared to cattle as the reference species (Table 2B and Figure 2). No significant differences were observed between goats and sheep in the *post-hoc* analysis. This suggests that small ruminants are significantly more likely to be seropositive than cattle and potentially generate a higher risk of infection when compared to the latter species. To further investigate these trends, we examined the significance of herd composition, namely herds with single- or mixed-species. Herds that included only small ruminants (OR = 6.54, CI [3.65–11.72]) or a mix of cattle and small ruminants (OR = 2.96, CI [1.90–4.60]) had a significantly higher OR of carrying seropositive animals compared to herds with only cattle as the reference unit (Table 2C). Significant differences in ELISA seropositivity were also found between mixed and only small ruminant herds in the *post-hoc* contrast analysis (OR = 0.45, S.E. = 0.11, *p* = 0.0014). All together these findings suggest that the presence of small ruminants in herds can be a significant risk factor for seropositivity in livestock.

Collectively, these findings suggest that the specific herd, as a unit, and its animal species composition are robust predictors for brucellosis seropositivity and thus potential risk factor of infection. To provide a visual overview of this pattern, we summarized in Figure 1B the species composition and related seroprevalence within each sampled herd mapped in the ranch based on GPS coordinates, as recorded in EpiCollect (35). This mapping confirmed the previous findings (Tables 1A, 2A and Supplementary Table S1), moreover, it illustrates that there is a tendency for the seroprevalence of herds with only cattle to be higher when in close proximity of herds with small ruminants, for example compare Herd A with Herds I.

During the structured interviews, the ranch management supplied approximate counts of cattle, goats and sheep within their herds. These data showed that while the probability of seropositive cases was significantly associated with abundance of each species, the odd ratios were around 1 (OR[CI]: cattle = 0.997[0.996–0.997]; goats = 1.002[1.001–1.003] and sheep = 0.991[0.987–0.996]; for all: *p* < 0.001). Specifically, herds size is a significant but small risk factor for cattle, sheep (with larger farms being at less risk) and goats (with larger farms presenting an increased risk). This finding supports the hypothesis that herd size plays a crucial role in facilitating the endemic circulation of *Brucella* and this seems to be more significant for goats than for cattle or sheep.

We finally examined if ELISA seropositivity was associated with different animal characteristics, either age, sex or female lactation status, independently for each species. Seroprevalence was high in both old (>2 yrs) and young (7 m-2 yrs) goats (~69%), while it was lower for cattle in both age classes (~21%), values were higher in old (71.4%, CI [52.11–90.75]) than young sheep, though the number of animals in the latter group was small (Table 1B). Yet, the logistic regression indicated that age was a highly significant risk factor for cattle (Table 2D), where older animals are more likely to test seropositive compared to younger animals (OR = 5.23, CI [2.70–10.10]); age was not significant for small ruminants.

When the sex was considered, seroprevalence was high in small ruminants compared to cattle in both females and males (Table 1C). Specifically for each species, the percentage was higher in female goats (70.9%, CI [63.43–78.42]) and male sheep (66.7%, CI [42.81–90.52]) but it was generally low in both cattle sexes (7.8–22.9%). Animal sex was not statistically significant in any of the three livestock species, suggesting that it might have a minor role on brucellosis seroprevalence.

Data on lactating animals were collected for cattle and goats, whereas no sheep were found in such a status during sampling. Lactating goats had a seropositivity rate of 74.4%, and a significant positive odd ratio (OR = 3.65, CI [1.16–11.50]); this contrasted with the low seropositivity found in cattle (38.3%), although the odd ratio was significant (OR = 2.87, CI [1.78–4.63]) (Tables 1, 2). Lactation appears to be an important factor associated with the higher odds of brucellosis seropositivity in both species. The small sample size might have affected some of the analyses for sheep and result interpretation should provide general insights.

### Human serology

**Figure 3:**
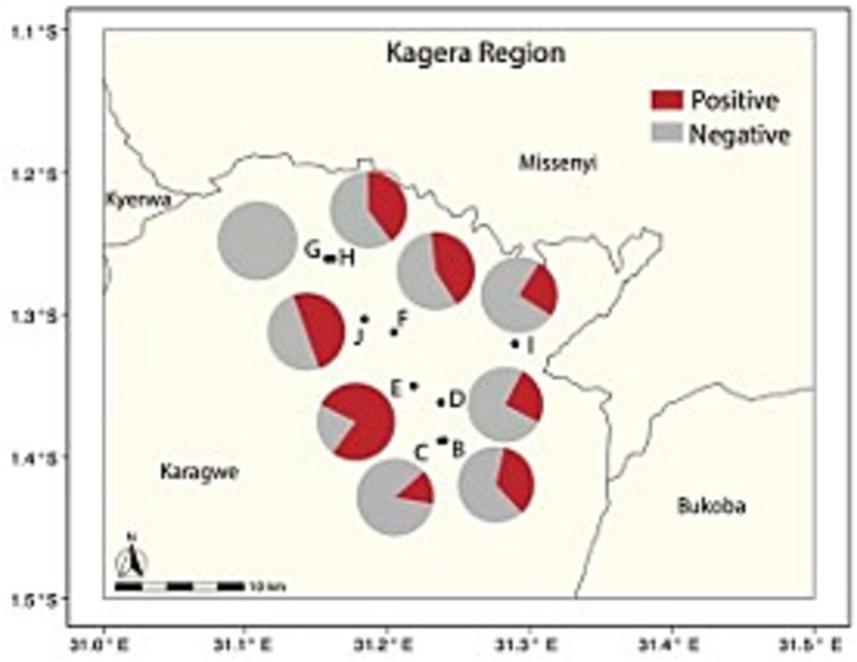
Percent seropositivity of humans from camps (pies) associated with livestock herds (circles and letters) in the ranch. Only the available human-livestock associations are considered.

We collected 112 human serum samples from residents of ranch camps, which included individuals who were in direct contact with animals (e.g., herders and animal health workers) or not directly involved (e.g., working or living around the ranch). The cELISA identified a rate of 41.1% seropositive cases (Table 3). To identify a potential spatial correlation between human and livestock seropositivity, human sera from camps adjacent to the corresponding herds and their composition were examined via GLM. Only the available adjacent camp ID -herd ID pairs were considered. A significant adjusted odd ratio was found when comparing seropositivity of human cases associated with Herd E (mixed species composition) and human cases related to the reference Herd B (only cattle) (OR = 6.56, CI [1.10–39.32]), all the remaining comparisons were not significant (Table 4A). A further *post-hoc* contrast analysis provided no significant human-herd pairwise associations. Similarly, no significant relationships were found between seropositive human cases and the livestock composition of the associated herd. These findings suggest that there is an overall weak spatial association in *Brucella* seropositivity between human camps and their reference herds. In other words, there is no clear evidence that human seropositivity is spatially structured and related to the distribution and composition of the herds. To facilitate the visualization of this spatial discordance (Figure 3) depicts the human seropositivity and can be compared with Figure 1B for livestock.

**Table 3:** Summary of seroprevalence data based on serum ELISA analysis from humans.

Human risk factors	N (Total, positive)	Seroprevalence % [CI]
A. Age		
18–25	(41, 18)	43.9 [28.71–59.09]
26–35	(40, 16)	40.0 [24.82–55.18]
36–45	(20, 4)	20.0 [2.47–37.53]
>46	(11, 8)	72.7 [46.41–99.05]
B. Sex		
Female	(15, 4)	26.7 [4.29–49.05]
Male	(97, 42)	43.3 [33.44–53.16]
C. Education level		
No school	(17, 10)	58.8 [35.43–82.22]
Primary school	(63, 25)	39.7 [27.60–51.76]
Middle or higher school	(32, 11)	34.4 [17.92–50.83]
D. Activities at risk		
Direct interaction with livestock	(85, 41)	48.2 [44.22–70.60]
Indirect interaction with livestock	(27, 5)	22.7 [6.23–36.63]
E. Livestock parturition and/or aborted fetuses		
No contact	(66, 25)	37.8 [26.18–49.58]
Contact	(46, 21)	45.7 [31.26–60.05]
F. Raw milk and/or meat		
No consumption	(36, 14)	38.9 [23.0–54.81]
Consumption	(76, 32)	42.1 [31.01–53.21]
Total	(112, 46)	41.07 [31.96–50.18]

**Table 4:** Logistic regression between serum ELISA (positive–negative) and potential risk factors for humans.

Risk factors	OR	95% CI	*p*-value
A. Herds (Reference: Herd B)			
Intercept	0.53	0.23–1.26	1.51E-01
Herd C	0.38	0.04–3.79	4.06E-01
Herd D	0.80	0.16–3.99	7.89E-01
**Herd E**	**6.56**	**1.10–39.32**	**3.94E-02**
Herd F	1.41	0.25–7.90	6.99E-01
Herd G	1.88	0.31–11.52	4.98E-01
Herd H	1.25	0.17–9.09	8.26E-01
Herd I	0.75	0.12–4.77	7.61E-01
Herd J	1.88	0.42–8.47	4.14E-01
AIC = 121.73; Accuracy = 0.73; Performance (AUC) = 0.74			
B. Activities at risk (Reference: no direct interaction with livestock)			
Intercept	**0.14**	**0.04–0.48**	**1.62E-03**
Direct interaction with livestock	**7.20**	**1.97–26.32**	**2.83E-03**
AIC = 120.50; Accuracy = 0.35; Performance (AUC) = 0.41			
C. Livestock parturition and/or aborted fetuses (Reference: no contact)			
**Intercept**	**0.5**	**0.29–0.86**	**1.14E-02**
**Contact**	**2.37**	**1.01–5.58**	**4.73E-02**
AIC = 128.64; Accuracy = 0.18, Performance (AUC) = 0.19.			

The Adjusted Odds Ratios (OR), Confidence Intervals (CI), Statistical Significance (*p* < 0.05), Goodness of model fit (AIC), from the model fitted to the training dataset, and Model Accuracy and performance [i.e., Area Under the Curve (AUC)], from the tested dataset, are reported. The intercept represents the reference entity. The significant attributes are represented in Bold.

To further explore the human-livestock interaction, data on human activities and habits were available from the structured questionnaire with camp residents. Individuals in regular contact with animals and involved in various practices, such as herding, milking, feeding and providing animal health care (i.e., veterinarians), exhibited a significantly higher odd ratio (OR = 7.20, CI [1.97–26.31]) when compared to individuals with no direct involvement in the daily management or care of livestock (Table 4B). Among the groups at risk, a further analysis based on individuals engaged in the many activities of animal parturition showed that those who were involved with animal parturition and/or management of aborted fetuses had a significantly higher odd ratio (OR = 2.37, CI [1.01–5.58]) than individuals who did not participate in these activities (Table 4C). Individuals not directly engaged in livestock routines, may still be exposed to animals and their products, like milk and meat sourced from these herds. When we examined the habit of drinking untreated milk (either from cow or goat) and eating raw meat, no significant association was found with human seropositivity. Finally, demographic factors like age, gender, and education level were not significantly associated with the likelihood of human seropositive cases. We are aware that variation of human samples may have affected some of the results.

### Malaria testing

Malaria testing was performed on human samples to confirm their malaria status, as a standard procedure in malaria-endemic areas of Tanzania, and to examine whether there was an association between cases positive to malaria and *Brucella*. Our results identified that only three serum samples out of 112 were positive (2.7%), and none of these tested positive for anti-*Brucella* antibodies.

## Discussion

### Overview of key findings

Our study of brucellosis seroprevalence in a Tanzanian ranch system reveals complex dynamics of infection among livestock and human populations. To understand how variables representative of the livestock population contributes to local pathogen circulation and to what extent they are a risk to public health, we examined the seroprevalence of brucellosis among herds and the human communities managing these herds in a ranch system from the Kagera region. Key findings include significant variation in ELISA seropositivity among herds, with small ruminant-only and mixed herds showing higher seropositivity than cattle-only herds. Older cattle as well as lactating females of both cattle and goats were found to be significant risk factors for brucellosis seropositivity.

Contrary to expectations, spatial variation in human seropositivity from camps was not associated with spatial variation in herd seropositivity. Instead, human infection risk was linked to specific animal handling practices, particularly during parturition and management of aborted fetuses. Surprisingly, consumption of raw meat and/or milk did not significantly increase the number seropositive individuals, contrary to findings from previous studies (22, 43–46).

### Herd composition and *Brucella* transmission

The marked variation in seropositivity among herds suggests that herd composition and management practices play crucial roles in *Brucella* transmission. While *Brucella* is endemic throughout the region, our findings suggest that herds containing small ruminants, either exclusively or mixed with cattle, contribute to significantly higher seroprevalence compared to cattle-only herds. This pattern likely contribute to increased local transmission and a higher risk of infection for both animals and humans in close contact. We can speculate that the presence of small ruminants appears to be a critical factor in maintaining and potentially amplifying *Brucella* circulation within this ranch ecosystem, possibly via *Brucella melitensis*, known for its higher virulence in small ruminants and humans compared to *Brucella abortus* (47). Our finding underscores the need for targeted interventions in mixed herds and those with small ruminants, including agropastoral and pastoral systems of Tanzania, where the significance of small ruminants in the epidemiology of brucellosis has been noted (48, 49).

The lack of a significant spatial correlation between human and livestock seropositivity highlights the complexity of *Brucella* transmission in ranch settings. This pattern suggests that while animal-to-animal transmission maintains infection within herds, human infections may result from periodic exposure across multiple herds due to livestock management in the ranch. Indeed, this finding emphasizes the need for comprehensive surveillance and control strategies that consider both animal and human populations simultaneously.

### Human infection risk and livestock management practices

We found that the probability of human infections is affected by the type of activity and its level of risk once exposed to an infected animal. In the ranch, management practices can vary between herds, with each herd being managed independently according to its needs. Previous investigations have reported the presence of brucellosis in livestock from diverse production systems and geographical areas of Tanzania (14, 50–52). The ranch we investigated involves extensive livestock raised in large areas, which contrasts with small family farms or local pastoral and agro-pastoral settings or intensive farming systems (18, 53). It is possible, that the management of extensive ranches, combined with the proximity of human communities living on the ranch, whether for work or well-being, could contribute to *Brucella* persistence in livestock and infection in humans from the local or distal herds. In the absence of surveillance and control measures, the disease could spread to the neighboring regions through the movement of livestock and increases the likelihood of zoonotic transmission (10, 16, 54). Indeed, brucellosis is increasingly becoming a concern for small-scale dairy operations in semi-urban areas and for those that practice intensive farming (55). Our findings are consistent with the general observation that herd size is an important risk factor for *Brucella* infection of livestock, especially for small ruminants, and can contribute to pathogen endemicity.

### Age as a risk factor in cattle

Age emerged as a crucial risk factor in cattle, with older animals showing significantly higher odds of seropositivity. This higher seroprevalence in older animals might be expected and aligns with the notion that prolonged exposure to *Brucella* pathogens increases the likelihood of seroconversion (56, 57). The increased seroprevalence of *Brucella* infection in older cattle is in agreement with previous studies (58), where age was indicated as a notable risk factor for *Brucella* seropositivity in livestock populations. This observation holds potential implications for the long-term health and productivity of a herd, factors of paramount importance to Tanzanian farmers who frequently operate within constrained profit margins.

### Lactation status and brucellosis risk

The status of lactation in livestock, was identified as a risk factor for both cattle and goats. Lactating animals may be more susceptible to infection, which is probably associated with the physiological demands of lactation, including changes in hormonal and immune functioning and social behavior of female animals. Our finding has important implications for milk production, quality, and safety in rural Tanzania, where dairy products are crucial for nutrition and income. The infection risk associated to lactation could also be linked to recent parturition events, as livestock pregnancy has been observed to promote the proliferation of *Brucella* bacteria within ruminant hosts as well as the development of anti-*Brucella* antibodies, a pattern likely due to hormonal changes and alterations in the immune response during gestation (59). Similar conclusions were provided by other studies (60, 61), indicating that it is critical to consider the lactation status of livestock when evaluating the risk of brucellosis seropositivity and appropriate livestock management practices for disease control. However, when we examined the risk of seropositivity associated with the human consumption of raw milk and meat we found no significant relationship. More work is needed to clarify the role of raw food on the risk of *Brucella* infection, as consumption of contaminated animal products was suggested to be an important risk factor in other studies (62).

### Occupational risks and control measures

Our study confirms previous findings on occupational risks associated with animal handling (54, 63–65), particularly during parturition and management of aborted fetuses. The significance of proper personal protective equipment (PPE) use and safe animal handling practices cannot be overstated, especially in resource-limited settings.

The knowledge of these practices and, more broadly, of zoonotic infections that might pose a local threat can be an effective and relatively economical first approach to control *Brucella* and other zoonoses, and more should be done to improve fundamental awareness. This aligns with observations from other African countries, such as South Africa, where despite implemented control measures, brucellosis prevalence in cattle remained relatively stable from 2014 to 2019, suggesting the need for more comprehensive and consistently applied control strategies (66).

### One health approach and policy implications

The One Health approach, as emphasized in Tanzania’s national plan (67), is critical for addressing brucellosis. Our findings support the need for integrated surveillance and control measures that span both animal and human health sectors. This is particularly important given the potential impact of brucellosis on livestock productivity and international trade, which have significant economic implications for Tanzania and other LMICs. The success of brucellosis eradication in countries like the United States, achieved through rigorous surveillance, testing, and culling programs, stands in stark contrast to the ongoing challenges faced by countries in the Nile River Basin, including Tanzania (68). This disparity underscores the need for sustained, well-funded, and comprehensive control efforts in LMICs.

### Study limitations and future research directions

Our study offers valuable insights into brucellosis epidemiology within a Tanzanian commercial ranch system, but few limitations warrant consideration. The relatively modest human sample size may have affected our ability to detect significant associations within the human population and when examining human data in relation to livestock patterns. The inherent heterogeneity of livestock management systems, environmental factors, and animal populations across Tanzania precludes direct extrapolation of our findings from this single commercial ranch to the broader national context of ranching operations. This also suggests cautious interpretation when considering the applicability of our results to diverse ranch ecosystems in other countries. Additionally, potential seasonality in individual longitudinal variation and interannual brucellosis transmission dynamics were not captured due to our sampling design. Despite these constraints, our research provides essential baseline understanding on brucellosis seroprevalence and associated risk factors in a commercial ranch setting. These findings contribute to the body of knowledge on brucellosis in East Africa, inform future research directions, and support the development of targeted control strategies for similar settings. Future studies encompassing a wider range of ranch types, geographical locations, and longitudinal monitoring would further enhance our understanding of brucellosis epidemiology in Tanzania and similar contexts.

## Conclusion

The distinct spatial distribution of *Brucella* seroprevalence between herds, not mirrored in human cases near these herds, suggests complex transmission dynamics in this Tanzanian ranch system. Our findings highlight the need for targeted, comprehensive strategies to control brucellosis at the human-animal interface. We highlighted where some of these non-linearities emerge and the factors that can generate variation in seroprevalence. These findings have important implications for herd management practices, particularly in settings where livestock productivity is crucial for local economies.

Some of our results support previous findings; however, further research is needed to confirm the factors and processes that affect *Brucella* seroprevalence across different agricultural settings and to clarify the epidemiology of transmission from livestock to humans.

Future research priorities should include longitudinal studies to understand seasonal variations and long-term transmission dynamics, investigations into the molecular epidemiology of *Brucella* species in this region, and evaluations of the cost-effectiveness of different control strategies. Finally, adopting a One Health approach by fostering collaboration between public health, veterinary, and environmental sectors is crucial for developing holistic control strategies. Establishing a One Health task force at the regional level could coordinate these efforts effectively. Implementation of these recommendations could significantly improve brucellosis control in Tanzania and similar settings, reducing both economic losses in the livestock sector and public health risks. Finally, future research should also focus on evaluating the effectiveness of these interventions and how they are altered by the many sources of heterogeneities that impact these systems.

## Data Availability

The raw data supporting the conclusions of this article will be made available by the authors, without undue reservation.
